# Supplementary material for: Non-equilibrium time-dependent solution to discrete choice with social interactions
Source: PLoS One. 2022 May 26;17(5):e0267083. doi: 10.1371/journal.pone.0267083 (PMC9135261; doi:10.1371/journal.pone.0267083)
Supplement: S1 File — (ZIP) [file pone.0267083.s001.zip › SI.pdf]

Supplementary Information:

Non-equilibrium time-dependent solution to discrete choice with social interactions

James Holehouse[1] and Hector Pollitt[2]

[1]School of Biological Sciences, University of Edinburgh,

[2]The World Bank.

April 11, 2022

1 1D master equation

In this section we derive the master equation for a single discrete stochastic variable n. There are many sources for this, but the primary sources we use for problems of this type are [4, 7]. First we introduce $W_{nn'}$ as the transition probability per unit time to transition from state n' to n, and hence the probability to transition from n' to n in a time interval Δt is $W_{nn'}\Delta t$. One can then write an equation for the conservation of probability at $t + \Delta t$,

$$\mathcal{P}(n, t+\Delta t) = \sum_{n' \neq n} \mathcal{P}(n', t) W_{nn'} \Delta t + \left(1 - \sum_{n' \neq n} W_{n'n} \Delta t\right) \mathcal{P}(n, t). \tag{1}$$

The first term on the right-hand side of this equation describes the flow of probability into the state n from all other states n' in $[t, t + \Delta t)$, whereas the second term describes the probability of remaining in state n in $[t, t + \Delta t)$. One can rearrange this, taking $\mathcal{P}(n, t)$ on the right-hand side to the left, and dividing everything by Δt. In the limit $\Delta t \to 0$ we then arrive at the 1D master equation,

$$\partial_t \mathcal{P}(n, t) = \sum_{n' \neq n} W_{nn'} \mathcal{P}(n', t) - \mathcal{P}(n, t) \sum_{n' \neq n} W_{n'n}, \tag{2}$$

subject to the initial condition $\mathcal{P}(n, 0) = \mathcal{Q}(n)$ for some general initial distribution $\mathcal{Q}(n)$ and the normalisation condition $\sum_n \mathcal{P}(n, t) = 1$. From the form of Eq. (2) one can see the master equation as a 'gain-loss' equation in the probability $\mathcal{P}(n, t)$, the first right-hand side term describing the gain and the second describing the loss [4]. For the derivation of Eq. (14) in the main text we identify,

$$W_{nn'} = \begin{cases} b_n, & n' = n+1, \\ a_n, & n' = n-1, \\ 0, & \text{otherwise}. \end{cases} \tag{3}$$

2 Time-dependent zeitgeist solution

In this section we derive the analytic solution to the mean-field binary decision model under time-dependent $F(t)$. Let $F(t)$ be a piecewise defined function up to the time of interest t_T, divided over T intervals of equal length, where each interval is constant, i.e., $F(t_{j-1} < t < t_j) = F_j$ for $j \in \{1, 2, ..., T\}$ and $t_0 = 0$. In the case where $F(t)$ is not piecewise defined it can be approximated to any degree of accuracy as a piecewise function where $F_j \approx F(t_{j-1})$ and the accuracy of the approximation is improved for increasing T. Then over each time interval the propensities $a_i^j(t)$ and $b_i^j(t)$ for interval j take constant values and hence the master operator in Eq. (15) (main text) is a constant matrix within each interval. The solution requires that in each of these intervals that the eigenvalues λ_i^j for interval j and $i \in \{1, 2, ..., N+1\}$ are calculated computationally. Note that the more piecewise elements of $F(t)$ there are the longer this will take computationally. For the first interval, given some initial condition $\mathbb{Q}_1(m(n))$ at $t = 0$, the solution in $0 < t < t_1$ is,

$$P_1(m(n), 0 \leq t < t_1 | \mathbb{Q}_1(m(n)), 0) = \sum_{n_0=0}^{N} \mathbb{Q}_1(m(n_0)) P_1(m(n), t | m(n_0), 0) \tag{4}$$

where $P_1(m(n), t|m(n_0), 0)$ is defined as in Eqs. (16)-(20) (main text) but with $\lambda_i \to \lambda_i^1$. For the next interval $t_1 < t < t_2$ the solution is very similar but we now use the initial condition $\mathbb{Q}_2(m(n)) = P_1(m(n), t_1|\mathbb{Q}_1(m(n)), 0)$, giving us,

$$P_2(m(n), t_1 \leq t < t_2 | \mathbb{Q}_2(m(n)), t_1) = \sum_{n_0=0}^{N} \mathbb{Q}_2(m(n_0)) P_2(m(n), t - t_1 | m(n_0), 0), \tag{5}$$

where one now uses $\lambda_i \to \lambda_i^2$ in Eqs. (16)-(20) (main text). One then repeats this process for all intervals $j \in \{3, 4, ..., T\}$ up to $P_T(m(n), t_{T-1} < t < t_T|\mathbb{Q}_{T-1}(m(n)), t_{T-1})$. This completes the solution. We note that this is a similar method to how time-dependent transcription rates are dealt with in Section 10 of the supplementary information of [9].

3 Stochastic simulation algorithm

The SSA, also known as the *Gillespie algorithm* named after the scientist who popularised its use, provides a popular Monte Carlo method to simulate the economic model considered in this paper [6,10]. The major benefit of the SSA is that, unlike the Metropolis-Hastings algorithm [3] or the Glauber update algorithm [5], the SSA provides a continuous time description of stochastic processes. In this paper we use the SSA to simulate stochastic trajectories for systems of economic agents based on the transition probabilities for each agent to change their decision in Eq. (9) (main text). Below we will re-introduce some of the formulae previously presented in the paper for the reader's benefit. Note that although we use the SSA in this context to simulate trajectories from the mean-field binary decision model, one can use the same method for the more generalised system of non mean-field agents, agents with personal preferences described at the start of Section 2 (main text).

Consider a system of mean-field economic agents described in Section 2 (main text). A given agent i at a time t will have made a decision $S_i \in \{-1, 1\}$, and at some future point in the future the agent can change their decision to $-S_i$. The rate at which any agent i will change their decision is given by,

$$W_n(S_i \to -S_i) = \frac{\gamma}{1 + \exp(-\beta \mathcal{G}_i)}, \tag{6}$$

which is dependent on the number of agents n already deciding for $S_i = 1$ and the gain function defined in Eq. (8) (main text). Now, since all the agents have the same influence $I(n,t)$ upon them, we can further define the total propensities with which any agent can change their decision from $1 \to -1$ or $-1 \to 1$, respectively,

$$f_n^+ = (N - n)W_n(-1 \to 1),$$
$$f_n^- = nW_n(1 \to -1).$$

These propensities follow intuitively from the law of mass-action since the rate at which agents change their decision from $1 \to -1$ is proportional to the number of right deciding agents n [2,6]. We denote the total propensity at which any decision changes are made as $f_n = f_n^+ + f_n^-$, and state the *fundamental premise* that in time interval $[t, t + \Delta t)$ the probability that *any* agent will change their decision is $f_n \Delta t$ [6]. It follows that agent decision changes are exponentially distributed, and the waiting time u for the next change of decision (of any type) given the current state of the system n is drawn from,

$$u \sim \frac{1}{f_n} \exp(-f_n u). \tag{7}$$

The probability that this decision change at $t + u$ is of type is $1 \to -1$ is f_n^- / f_n, and the probability that this decision change is $-1 \to 1$ is f_n^+ / f_n. One then samples which decision will change next from these probabilities and updates the number of left and right voting agents accordingly. *Note that this algorithm can only be used in the current form (and via the direct method below) where $F(t)$ does not have time dependence, in which case, modifications to the algorithm must be considered* [11, 12]. We now detail the pseudo-code for the SSA algorithm via the *direct method* (a computationally faster but identical approach to the more intuitive algorithm above) [6]:

1. For $t = 0$, initialise the state variable of the system defined by the number of right voting agents n in a population of N agents. As each agent has a randomised initial choice with probability p of deciding right, n is drawn from a binomial distribution, i.e., $n \sim \text{Bin}(N, p)$ (other initial conditions can be used). Define a $M + 1$ element $\vec{V}$ vector that stores the state of the system at times $\mathcal{T} = \{0, \delta t, 2\delta t, ..., M\delta t\}$, for some time step δt. Set $V_0 = n$.

2. Calculate f_n for the current state of the system n.

3. Draw 2 random numbers, r_1 and r_2, from the uniform distribution over the unit interval $[0, 1]$. The

waiting time, u, for the next decision change is then found to be,

$$u = \frac{1}{f_n} \ln\left(\frac{1}{r_1}\right).$$

4. Update the storage of the state of the system between $[t, t+u)$. For every element of index i in $\mathcal{T}$ such that $t \leq \mathcal{T}_i \leq t+u$ assign $V_i = n$. Then, update the system time as $t \to t+u$. If $f_n^+ > r_2 f_n$ then flip the decision of left voting agent to right, i.e., update $n \to n+1$, otherwise flip the decision of right voting left to right, i.e., update $n \to n-1$.

5. If $t > M\delta t$ stop the simulation and return $\vec{V}$, otherwise go back to step 2.

Steps 1-5 detail the SSA for a single trajectory. In order to calculate the probability state vector, mean and variance at the times $\mathcal{T} = \{0, \delta t, 2\delta t, ..., M\delta t\}$ an ensemble of simulations would need to be produced. Say one produces an ensemble of E simulations each of which outputs a state vector $\vec{n}_i,\ i \in [1, 2, ..., E]$ of length $M+1$, where $\vec{n}_i$ is the state vector over all times in $\mathcal{T}$ for ensemble simulation i. Note that due to the stochastic nature of the SSA, each trajectory in the ensemble will be different from each other *even when the initial conditions of each simulation are the same.* The mean and variance at each time point, over the ensemble of simulations is given by,

$$\langle n((m-1)\delta t)\rangle = \frac{1}{E}\sum_{i=1}^{E}[n_i]_m, \tag{8}$$

$$\mathrm{Var}(n((m-1)\delta t)) = \frac{1}{E}\sum_{i=1}^{E}[n_i]_m^2 - \langle n((m-1)\delta t)\rangle^2, \tag{9}$$

for $m \in [1, 2, ..., M+1]$, where $[n_i]_m$ is the mth measurement of the ith trajectory. The probability distribution $\mathcal{P}(n,t)$ is instead given by the normalised histogram over each time slice in the ensemble. Explicitly this is,

$$\mathcal{P}(n,(m-1)\delta t) = \frac{\#\text{ of times } n \text{ appears in } [n_i]_m,\ \forall\, i \in [1,2,...,E]}{E}. \tag{10}$$

One can verify that for each slice of time the probability distribution is indeed normalised, i.e., $\sum_n \mathcal{P}(n,t) = 1$.

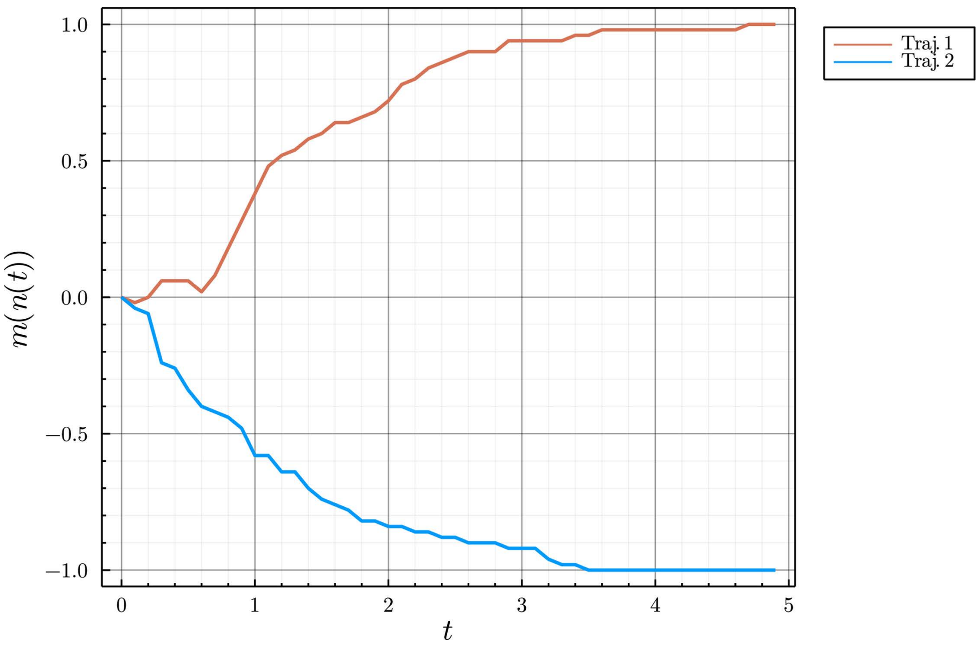

SI Figure 1: For the parameters shown in Fig. 1(c) in the main text there are two associated trajectories that the system can undertake. Either the SSA trajectory will show a steady evolution to $m(N) = 1$ or else it will evolve to $m(0) = -1$. These two realisations of the process occur with the same probability for $F = 0$ and $J > 1/\beta(\alpha + 1)$.

4 Criticality in the mean-field model

In this section we explore some interesting features of the mean-field model relating to critical behaviour. For $F = 0$ this allows us to identify a critical rationality of the agents β_c where for any $\beta > \beta_c$ we observe the occurrence of a bimodal steady-state distribution for $m(n)$. First though, it is useful to have a deterministic description of the dynamics such that one can identify the possible equilibrium values of m for a given parameter set. From the dynamics of Eq. (12) (main text) we write the deterministic rate equation as follows,

$$\partial_t \langle n \rangle = (N - \langle n \rangle) r(\langle n \rangle) - \langle n \rangle l(\langle n \rangle), \tag{11}$$

where $r(n)$ and $l(n)$ are rates defined in the main text. Solving this at steady-state in the limit $N \to \infty$ one can show,

$$m = \tanh\left(\beta(F + J(1 + \alpha)m)\right) \tag{12}$$

This equation is transcendental and one must identify the solutions to it computationally. It is interesting to ask how many solutions of m one expects for different values of the parameters. Consider setting the zeitgeist to zero, $F = 0$, where the only influence on the agents is now from the mean-field interactions. For there to be 3 solutions (2 stable, 1 unstable determined by the Jacobian of Eq. (11)) of this equation one sees that the gradient of the right-hand side of Eq. (12) with respect to m must be greater than 1. To leading order in an expansion in m, one can identify a critical value of agent rationality, $\beta_c = 1/J(\alpha+1)$, for which any small increase above β_c leads to 3 solutions of Eq. (12) and two stable equilibrium values of m. One can see this from the top left and central plots in SI Fig. 2, for $\beta = \beta_c$ there is 1 intersection point between the two sides of the equation, whereas for $\beta \gtrsim \beta_c$ there are 3 intersection points, with the middle intersection point being unstable. This is exhibited in the analytical solution from Eq. (16) (main text) in the bottom left and central plots: for $\beta = \beta_c$ the steady-state distribution has a very flat top and any small increase in β leads to a bimodal steady-state distribution. This bimodal behaviour is seen in individual realisations of the SSA in SI Fig. 1: for $\beta > \beta_c$ agent behaviour takes one of two directions, they either drift to mostly deciding right *or* left.

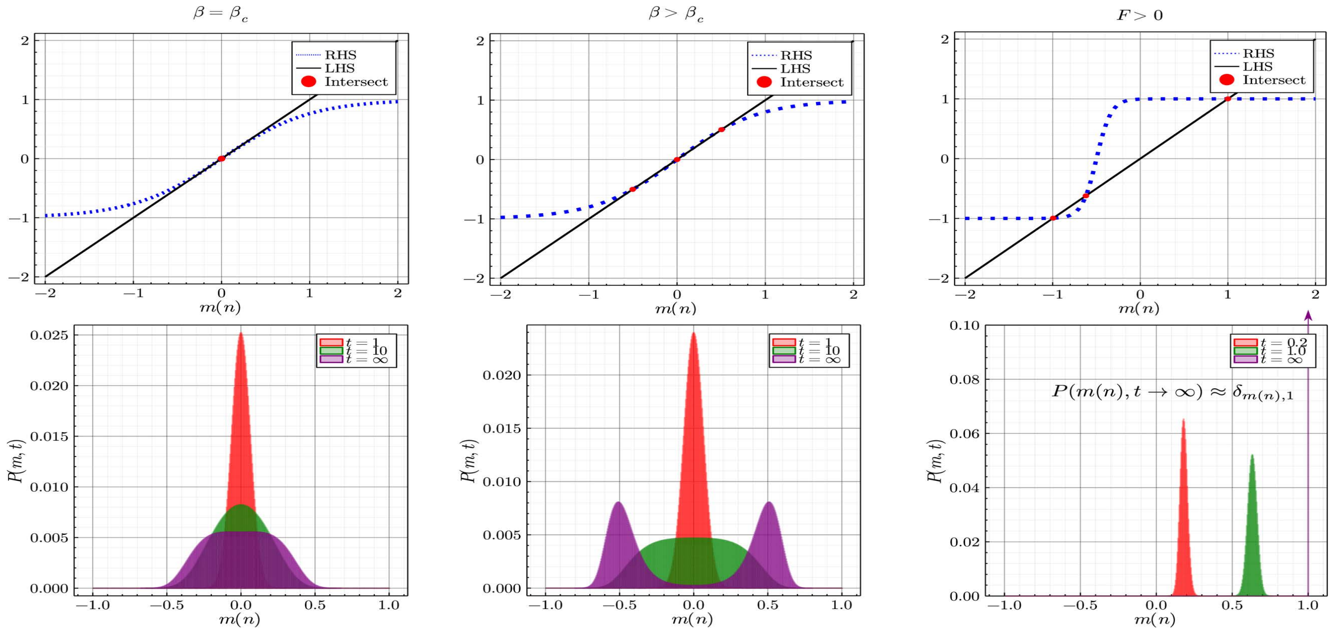

SI Figure 2: Plots show the critical behaviour of the mean-field model for $N = 500$. The top row of plots shows the solutions of the equation $m = \tanh(\beta(F + J(\alpha+1)m))$ as red points for different values of β. LHS denotes the left-hand side of this equation and RHS denotes the right-hand side. The bottom row of plots shows the evolution of the probability distributions over $m(n)$ from near the initial condition to the steady-state, obtained from Eqs. (16) and (22) (main text), and correspond to plots in the row above them. Parameters for column 1 are: $J = 1, \alpha = 0, F = 0$ and $\beta = 1$. Parameters for column 2 are: $J = 1, \alpha = 0, F = 0$ and $\beta = 1.1$. Parameters for column 3 are: $J = 1, \alpha = 0, F = 0.5$ and $\beta = 6$.

Finally, one can consider what happens for $F \neq 0$, which is explored in the final column of SI Fig. 2. In some situations we find three solutions to Eq. (12), in other cases only 1. However, even where 3 solutions are found (as in SI Fig. 2) the agents are exponentially more likely to favour being in the configuration where their decision has the same sign as the sign of F. This is seen in the bottom right plot in SI Fig. 2 for $F > 0$, even though there exists a second stable solution at $m \approx -1$, as $t \to \infty$ the

steady-state is $P(m, t \to \infty) \approx \delta_{m,1}$. This exponential behaviour is further supported by Eqs. (27)-(28) in the main text.

5 Derivation of ϕ_R

In this section we derive ϕ_R, the probability to end up with the agents coalescing on the right-hand technology given they start at n_u (or equivalently m_u with respect to the order parameter). Note that, for the sake of repetition the reader may already want to have read Section 3.2.2 in the main text, since we reference necessary equations from that section in the derivation below. We calculate ϕ_R in the same way that fixation probabilities are calculated for birth-death processes with two absorbing boundaries (see [4,8]). As stated in the main text, we calculate the 'equilibrium values' of n based on the extrema of the steady-state probability distribution. In the case where we satisfy the conditions set out in Section 3 (main text) and SI Sec. 6 ($\beta > \beta_c$ and $|F| < J(1+\alpha)$) there will be three extremal values, the middle one corresponding to the unstable equilibrium n_u and the other two, $n_- < n_u$ and $n_+ > n_u$, being the stable modes of the bimodal agent behaviour (i.e., the stable equilibrium points).

Now for the calculation of ϕ_R we must consider a separate microstate diagram to the one explored in Fig. 1(b) (main text), in particular n_- and n_+ become absorbing states, as shown in SI Fig. 3(a). The question we now ask is given that one starts at some n in $n_- < n < n_+$, what is the probability ϕ_i of getting fixated at the right-hand mode? Note that in our notation $\phi_R \equiv \phi_{n_u}$. The value of $1 - \phi_R$ then gives the probability of getting fixated at the left-hand mode. To proceed we see that $\phi_i = \lim_{t\to\infty} Q_{n_+,i}(t)$ from Eq. (33) (main text), i.e., the probability of being found at $n = n_+$ as $t \to \infty$, given one start at $t = 0$ at $n = i$, for the microscopic transitions in SI Fig. 3(a). Hence, we get the following recursive equation for ϕ_i,

$$\phi_i = a_{i+1}\Delta t \phi_i + b_{i-1}\Delta t \phi_{i-1} + (1 - (a_{i+1} + b_{i-1})\Delta t)\phi_i, \tag{13}$$

where we note that by definition $\phi_{n_-} = 0$ and $\phi_{n_+} = 1$. To solve this equation we introduce the difference variable $\nu_i = \phi_i - \phi_{i-1}$, which converts this equation into,

$$\nu_i = \frac{b_{i-2}}{a_i}\nu_{i-1}. \tag{14}$$

Solving this equation recursively then gives,

$$\nu_i = \left(\prod_{j=n_-+1}^{i-1} \frac{b_{i-1}}{a_{i+1}}\right)\phi_{n_-+1}, \tag{15}$$

since $\nu_{n_-+1} = \phi_{n_-+1}$. In order to find ϕ_{n_-+1} one can then show that $\sum_{k=n_-+1}^{n_+} \nu_k = \phi_{n_+} = 1$ and hence

we find,

$$\phi_{n_-+1} = \left(1 + \sum_{k=n_-+1}^{n_+-1} \prod_{j=n_-+1}^{k} \frac{b_{i-1}}{a_{i+1}}\right)^{-1}. \tag{16}$$

Finally, we can calculate all the ϕ_i through,

$$\phi_i = \sum_{k=n_-+1}^{i} \nu_k = \frac{1 + \sum_{k=n_-+1}^{i-1} \prod_{j=n_-+1}^{k} \frac{b_{i-1}}{a_{i+1}}}{1 + \sum_{k=n_-+1}^{n_+-1} \prod_{j=n_-+1}^{k} \frac{b_{i-1}}{a_{i+1}}}. \tag{17}$$

This completes our derivation of $\phi_R = \phi_{n_u}$. In SI Fig. 3(b) we plot the fixation probability ϕ_i for the parameters of Fig. 3 in the main text, and find $\phi_u \sim 0.534$. This is expected, since $F > 0$ there a greater chance of first hitting the right boundary than the left one.

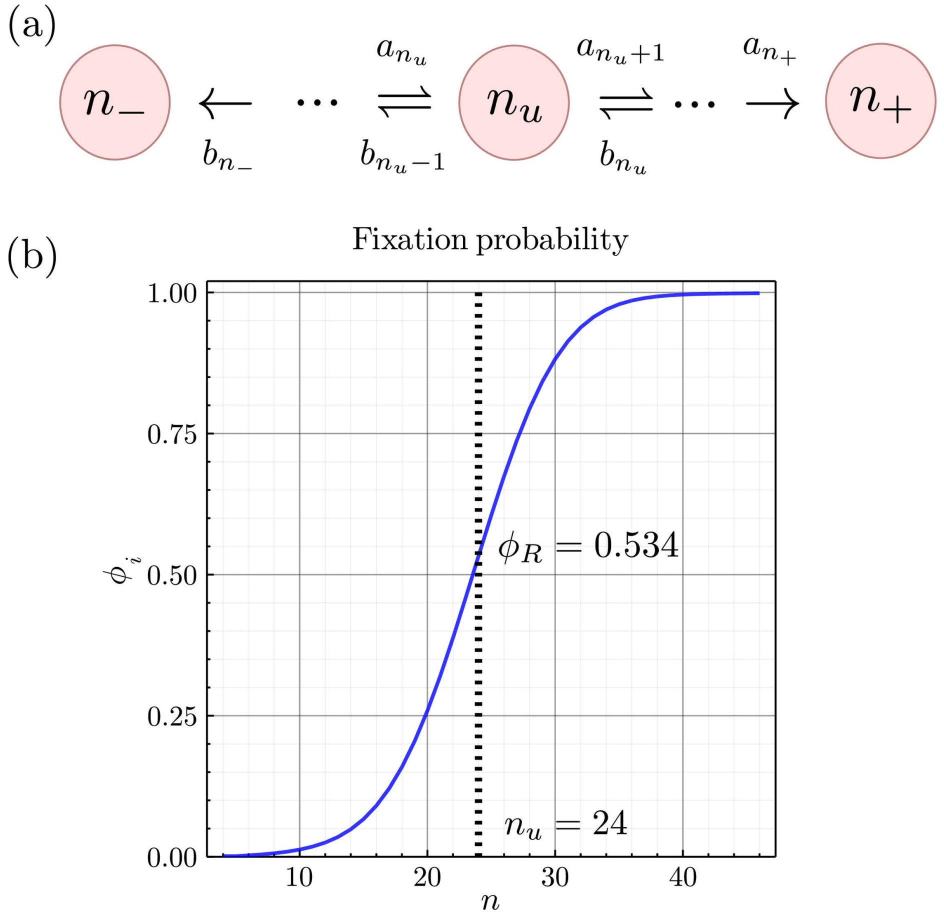

SI Figure 3: Plots for the calculation of ϕ_R. (a) We calculate ϕ_R as a fixation probability to end up with a system of agent coalesced on the right technology by artificially imposing absorbing boundaries at the modes of the behaviour of the agents. From this artificial system ϕ_R is well defined. (b) Plot of the fixation probability for the same parameters as Fig. 3 (main text). For this parameter set we find the equilibria values from the steady-state distribution are $n_- = 3$, $n_+ = 47$ and $n_u = 24$ (noting $N = 50$ and $F > 0$). Clearly since $F > 0$ it makes sense that at the unstable equilibrium there is a slightly greater chance of end up at the right technology which is optimal for the agents.

6 Derivation of approximate relaxation time scales

In this section we derive Eqs. (27) and (28) in the main text. Following the notation of [1] we define $\phi = n/N = (m+1)/2$, which is the fraction of agents choosing the right technology. Using the results from the standard textbooks [4, 7], one can approximate the master equation in Eq. (14) (main text) by a Fokker-Planck equation (FPE),

$$\partial_t \mathcal{P}(n,t) = -\partial_n[((N-n)r(n) - nl(n))\mathcal{P}(n,t)] + \frac{1}{2}\partial_n^2[((N-n)r(n) + nl(n))\mathcal{P}(n,t)], \tag{18}$$

where n is now a continuous variable $\in (-\infty, \infty)$. The FPE is a good approximation of the original master equation if $N \gg 1$ and $r(n)$ and $l(n)$ are smooth, slowly varying functions with respect to continuous n. One can then change variables from $n \to \phi$ to arrive at the following FPE now in ϕ,

$$\partial_t \mathcal{P}(\phi,t) = -\partial_\phi(a_1(\phi)\mathcal{P}(\phi,t)) + \frac{1}{2N}\partial_\phi^2(a_2(\phi)\mathcal{P}(\phi,t)), \tag{19}$$

where $\partial_n = N^{-1}\partial_\phi$ and we have defined,

$$a_1(\phi) = \gamma\left((1-\phi) - \left(1 + e^{\beta f(\phi)}\right)^{-1}\right), \tag{20}$$

$$a_2(\phi) = \gamma\left((1-\phi) + (2\phi - 1)\left(1 + e^{\beta f(\phi)}\right)^{-1}\right), \tag{21}$$

with,

$$f(\phi) = 2(F + J(\alpha+1)(2\phi - 1)). \tag{22}$$

Note that since $N \gg 1$ we have ignored the negligible self interaction term. In the limit $N \to \infty$ the noise term disappears entirely and the FPE equation reduces to a deterministic description via a rate equation in the mean value $\langle\phi\rangle$. Multiplying Eq. (19) by ϕ, setting the noise term to zero and integrating over all ϕ we arrive at the rate equation,

$$\partial_t\langle\phi\rangle = \langle a_1(\phi)\rangle \approx a_1(\langle\phi\rangle), \tag{23}$$

with the steady-state value(s) of ϕ determined by $a_1(\langle\phi\rangle_s) = 0$. Note that the approximation $\langle a_1(\phi)\rangle \approx a_1(\langle\phi\rangle)$ is valid so long as the fluctuations of ϕ about $\langle\phi\rangle$ are small compared to $\langle\phi\rangle$. For $\beta > \beta_c$ and $|F| < J(1+\alpha)$ there are three equilibrium solutions of $\langle\phi\rangle_s$: two of which are stable $\langle\phi\rangle_s = 0, 1$ and one of which is unstable $\langle\phi\rangle_u = \frac{1}{2}(1 - \frac{F}{J(\alpha+1)})$. If either of these conditions are broken then there is only one equilibrium solution. Our interest now is to find mean first passage times of getting to the unstable equilibrium point, starting from each of the stable equilibria.

Again, following standard methods for the FPE [4], one can find the mean first passage time to reach ϕ_u given initially at ϕ as,

$$\tau_\phi = 2N \begin{cases} \int_\phi^{\phi_u} e^{\Phi(y')}dy' \int_0^{y'} e^{-\Phi(y'')}\frac{dy''}{a_2(y'')}, & \phi < \phi_u, \\ \int_{\phi_u}^{\phi} e^{\Phi(y')}dy' \int_{y'}^{1} e^{-\Phi(y'')}\frac{dy''}{a_2(y'')}, & \phi > \phi_u. \end{cases} \tag{24}$$

where we have further defined,

$$\Phi(y) = -2N\int_0^y \frac{a_1(y)}{a_2(y)}dy. \tag{25}$$

In order to proceed we investigate the limit $\beta \to \infty$, i.e., where agents are highly rational. In this limit one can show that,

$$\frac{a_1(y)}{a_2(y)} \sim \begin{cases} -1, & y > \phi_u, \\ 0, & y = \phi_u, \\ 1, & y < \phi_u. \end{cases} \tag{26}$$

This then gives us a simplified form of $\Phi(y)$ in the highly rational limit,

$$\Phi(y) \sim \begin{cases} 2Ny, & y \le \phi_u, \\ 2N(2\phi_u - y), & y \ge \phi_u. \end{cases} \tag{27}$$

$\Phi(y)$ is now approximately a triangular function with its maximum at ϕ_u and minima at 0 and 1. Since the function is peaked around ϕ_u one can then successively use two saddle point approximations on

Eqs. (24) [13]. Doing so gives our required mean first passage times in the limit $\beta \to \infty$,

$$\tau_{lr} = \tau_0 \sim \frac{2\pi}{\sqrt{\Phi''(0)|\Phi''(\phi_u)|}} \exp\left(N\left(1 - \frac{F}{J(\alpha+1)}\right)\right), \tag{28}$$

$$\tau_{rl} = \tau_1 \sim \frac{2\pi}{\sqrt{\Phi''(1)|\Phi''(\phi_u)|}} \exp\left(N\left(1 + \frac{F}{J(\alpha+1)}\right)\right), \tag{29}$$

where the double prime denotes the second derivative of $\Phi(y)$ from Eq. (25). Note that shown here is the full result of the calculation, whereas in the main text we show only the proportionality to the exponential function. As one would expect from symmetry, if $F = 0$ then $\tau_{lr} = \tau_{rl}$ since $\Phi(y)$ becomes a symmetric function about ϕ_u.

References

[1] Bouchaud JP. Crises and collective socio-economic phenomena: simple models and challenges. Journal of Statistical Physics. 2013;151(3):567–606.

[2] Schnoerr D, Sanguinetti G, Grima R. Approximation and inference methods for stochastic biochemical kinetics—a tutorial review. Journal of Physics A: Mathematical and Theoretical. 2017;50(9):093001.

[3] Täuber UC. Critical dynamics: a field theory approach to equilibrium and non-equilibrium scaling behavior. Cambridge University Press; 2014.

[4] Van Kampen NG. Stochastic processes in physics and chemistry. vol. 1. Elsevier; 1992.

[5] Glauber RJ. Time-dependent statistics of the Ising model. Journal of mathematical physics. 1963;4(2):294–307.

[6] Gillespie DT. Stochastic simulation of chemical kinetics. Annu Rev Phys Chem. 2007;58:35–55.

[7] Gardiner C. Stochastic methods. vol. 4. Springer Berlin; 2009.

[8] Ashcroft P. Metastable States in a Model of Cancer Initiation. In: The Statistical Physics of Fixation and Equilibration in Individual-Based Models. Springer; 2016. p. 91–126.

[9] Cao, Zhixing and Grima, Ramon. Analytical distributions for detailed models of stochastic gene expression in eukaryotic cells. Proceedings of the National Academy of Sciences. 2020;117(9):4682–4692.

[10] Gillespie, D. Exact stochastic simulation of coupled chemical reactions. *The Journal Of Physical Chemistry*. **81**, 2340-2361 (1977)

[11] Anderson, D. A modified next reaction method for simulating chemical systems with time dependent propensities and delays. *The Journal Of Chemical Physics*. **127**, 214107 (2007)

[12] Thanh, V. & Priami, C. Simulation of biochemical reactions with time-dependent rates by the rejection-based algorithm. *The Journal Of Chemical Physics*. **143**, 08B601_1 (2015)

[13] Arfken, G. & Weber, H. Mathematical methods for physicists. (American Association of Physics Teachers,1999)
