# Supplementary material for: Non-equilibrium time-dependent solution to discrete choice with social interactions
Source: PLoS One. 2022 May 26;17(5):e0267083. doi: 10.1371/journal.pone.0267083 (PMC9135261; doi:10.1371/journal.pone.0267083)
Supplement: Peer review history

# Peer review: Decision Letter 0

**DOI:** 10.1371/journal.pone.0267083.r001

**Authors:** Talib Al-Ameri

4 Apr 2022

Non-equilibrium time-dependent solution to discrete choice with social interactions

PONE-D-22-00146

Dear Dr. Holehouse,

We’re pleased to inform you that your manuscript has been judged scientifically suitable for publication and will be formally accepted for publication once it meets all outstanding technical requirements.

Within one week, you’ll receive an e-mail detailing the required amendments. When these have been addressed, you’ll receive a formal acceptance letter and your manuscript will be scheduled for publication.

An invoice for payment will follow shortly after the formal acceptance. To ensure an efficient process, please log into Editorial Manager at http://www.editorialmanager.com/pone/, click the 'Update My Information' link at the top of the page, and double check that your user information is up-to-date. If you have any billing related questions, please contact our Author Billing department directly at authorbilling@plos.org.

If your institution or institutions have a press office, please notify them about your upcoming paper to help maximize its impact. If they’ll be preparing press materials, please inform our press team as soon as possible -- no later than 48 hours after receiving the formal acceptance. Your manuscript will remain under strict press embargo until 2 pm Eastern Time on the date of publication. For more information, please contact onepress@plos.org.

Kind regards,

Talib Al-Ameri, Ph.D

Academic Editor

PLOS ONE

Additional Editor Comments (optional):

I received the flowing comment from reviewer #2

The paper looks interesting however the following revision is needed.

1. Some quantitative results should be added to the abstract. The main findings should be highlighted in this section.

2. The number of pages too much large should be reduced to 20-25.

3. The author should explain the citation as mentioned [16-18], [19-23] etc.

I would consider these comments as optional comment.

Reviewers' comments:

Reviewer's Responses to Questions

**Comments to the Author**

1. Is the manuscript technically sound, and do the data support the conclusions?

The manuscript must describe a technically sound piece of scientific research with data that supports the conclusions. Experiments must have been conducted rigorously, with appropriate controls, replication, and sample sizes. The conclusions must be drawn appropriately based on the data presented. 

Reviewer #1: Yes

**********

2. Has the statistical analysis been performed appropriately and rigorously? 

Reviewer #1: Yes

**********

3. Have the authors made all data underlying the findings in their manuscript fully available?

The PLOS Data policy requires authors to make all data underlying the findings described in their manuscript fully available without restriction, with rare exception (please refer to the Data Availability Statement in the manuscript PDF file). The data should be provided as part of the manuscript or its supporting information, or deposited to a public repository. For example, in addition to summary statistics, the data points behind means, medians and variance measures should be available. If there are restrictions on publicly sharing data—e.g. participant privacy or use of data from a third party—those must be specified.

Reviewer #1: Yes

**********

4. Is the manuscript presented in an intelligible fashion and written in standard English?

PLOS ONE does not copyedit accepted manuscripts, so the language in submitted articles must be clear, correct, and unambiguous. Any typographical or grammatical errors should be corrected at revision, so please note any specific errors here.

Reviewer #1: Yes

**********

5. Review Comments to the Author

Please use the space provided to explain your answers to the questions above. You may also include additional comments for the author, including concerns about dual publication, research ethics, or publication ethics. (Please upload your review as an attachment if it exceeds 20,000 characters)

Reviewer #1: In the current work, the authors solved the binary decision model of Brock

and Durlauf in time using a method reliant on 9 the resolvent of the master

operator of the stochastic process. The method used for this solution can be

easily extended to other binary decision models, including Kirmans model of ant

recruitment, and under reinterpretation also provides a time-dependent solution

to the mean-field Ising model. Further, they used the time-dependent solution

to construct a likelihood function that can be used on non-equilibrium data for

model calibration.

The Abstract appropriately summarize the manuscript, there are no discrepancies between the Abstract and the remainder of the manuscript and the

Abstract can be understood without reading the manuscript.

The results clearly explained, their order of presentation is parallel to the

order of presentation of the methods. Also, the results, all reasonable and expected, are introduced and preceded by an appropriate and concise discussion

in the section.

The paper is of more than reasonable quality and novelty.

The conclusions of the authors are justified by the results found in the study.

The results are new and innovative and are considered a good addition to

previous works in the same field of work.

I suggest the acceptance of the paper in its present form for publication

in PLOS ONE.

**********

6. PLOS authors have the option to publish the peer review history of their article (what does this mean?). If published, this will include your full peer review and any attached files.

If you choose “no”, your identity will remain anonymous but your review may still be made public.

**Do you want your identity to be public for this peer review?** For information about this choice, including consent withdrawal, please see our Privacy Policy.

Reviewer #1: **Yes: **Mohamed I. Abbas

# Editor report: Acceptance letter

**DOI:** 10.1371/journal.pone.0267083.r002

**Authors:** Talib Al-Ameri

12 Apr 2022

PONE-D-22-00146 

Non-equilibrium time-dependent solution to discrete choice with social interactions 

Dear Dr. Holehouse:

I'm pleased to inform you that your manuscript has been deemed suitable for publication in PLOS ONE. Congratulations! Your manuscript is now with our production department. 

If your institution or institutions have a press office, please let them know about your upcoming paper now to help maximize its impact. If they'll be preparing press materials, please inform our press team within the next 48 hours. Your manuscript will remain under strict press embargo until 2 pm Eastern Time on the date of publication. For more information please contact onepress@plos.org.

If we can help with anything else, please email us at plosone@plos.org. 

Thank you for submitting your work to PLOS ONE and supporting open access. 

Kind regards, 

PLOS ONE Editorial Office Staff

on behalf of

Dr. Talib Al-Ameri 

Academic Editor

PLOS ONE
